# Multiple strategies were adopted to optimize the enzymatic characteristics and improve the expression of bovine chymosin BtChy in *Kluyveromyces lactis* for cheese production

**DOI:** 10.3389/fmicb.2025.1605229

**Published:** 2025-05-29

**Authors:** Ying Han, Liu-Qun Zhang, De-Ming Rao, Lei Lei, Jiang-Ke Yang

**Affiliations:** Pilot Base of Food Microbial Resources Utilization of Hubei Province, College of Life Science and Technology, Wuhan Polytechnic University, Wuhan, China

**Keywords:** calf chymosin, *in silico* engineering, signal peptide, promoter, gene dosage

## Abstract

Chymosin (EC3.4.23.4), primarily sourced from calf abomasum, serves as a conventional coagulant in milk curdling during cheese production. To improve the enzymatic properties and enhance the expression of calf chymosin (BtChy) in *Kluyveromyces lactis* to meet the demands of the cheese industry, the *in silico* engineering via hotspot scanning and molecular dynamics analysis was adopted. This approach improved the activity of BtChy on milk curdling and increased its sensitivity at 65°C. Multiple strategies were utilized to develop an environmentally friendly method for chymosin production. These included screening for constitutive promoters and signal peptides, as well as *in vitro* construction of a concatemer of the BtChy gene. The optimal combination, comprising the P_TDH3_ promoter, invertase signal peptide, and a four-copy BtChy gene integrated into the yeast genome, was identified. After high-density cultivation in a 5-L bioreactor, the recombinant yeast achieved an activity of 42,000 SU/mL, a 52.5-fold increase over the original wild-type chymosin gene.

## 1 Introduction

Cheese is one of the most popular foods, renowned for its rich nutrition, long shelf life, and exquisite, delightful flavor. Archeological relics, such as ancient pottery and rock paintings from the Neolithic era, suggest that the history of cheese-making dates back to around the 5th millennium BC (Brown and Pariser, 1975). Chymosin (EC 3.4.23.4), predominantly obtained from calf abomasum, has long-standingly functioned as an indispensable coagulant in the milk curdling process, thereby playing a pivotal role in cheese production (Johnson, 2017). Due to the scarcity of calf chymosin, since the 1960s, alternative sources of chymosin (proteinase) have been explored, including those from other young animals like lambs and camels (Mbye et al., 2021), fruit plants such as lemons and kiwis (Mazorra-Manzano et al., 2013), fungi (Yu et al., 1969), and bacteria (Meng et al., 2021). However, the strong protein hydrolysis activity of proteinases derived from plant and microbial sources often leads to extensive degradation of cheese caseins, imparting an unfavorable bitter flavor to the cheese. Consequently, calf chymosin remains the most preferred coagulant in cheese production (Kumar et al., 2010).

Milk comprises two primary protein groups: caseins and whey proteins. Caseins constitute 80% of milk’s total nitrogen content and primarily consist of four protein types: *α*-S1, α-S2, *β*, and *κ*. In milk, α-S1, α-S2, and β-caseins strongly bind to Ca^2+^, while κ-casein interacts with these to form colloidal particles. The *κ*-casein featureing a hydrophobic “hairy” C-terminus could prevent the aggregation and precipitation of the casein micelles, and stabilize the milk solutions (Pereira, 2014; Corredig et al., 2019). Calf chymosin demonstrates exceptional specificity for hydrolyzing κ-casein, cleaving the bond between Phe105 and Met106 in its “hairy” terminus. This action eliminates the repulsive forces between casein micelles, causing them to aggregate, precipitate from the solution, and form curd (Langholm Jensen et al., 2013).

Recombinant chymosin, serving as a substitute for traditional preparations derived from calf abomasum, offers several advantages, including a short growth cycle, high yield, low production costs, and a streamlined downstream extraction process. Heterologous expression of calf chymosin has been achieved in yeast (Vallejo et al., 2008; Feng et al., 2011), filamentous fungi (EFSA Panel on Food Contact Materials, Enzymes and Processing Aids (CEP), 2022), and bacteria (Menzella, 2011). In the 1990s, the U.S. Food and Drug Administration (FDA) approved recombinant calf chymosin expressed by *Escherichia coli* K-12, *Kluyveromyces marxians* var. *lactis*, and *Aspergillus niger*. Since then, the market demand for recombinant calf chymosin has steadily increased.

Strategies such as site-directed mutagenesis, ancestral sequence-based mutations, and redesigning of surface charges have been widely utilized to modify enzymatic properties (Reetz and Wu, 2008; Li et al., 2011; Sørensen et al., 2013; Zhang et al., 2019). Recently, the physics-based modeling methods such as molecular mechanics and quantum mechanics were used in computational enzyme engineering, and the tools such as SubTuner were also developed to predict beneficial enzyme mutants (Jurich et al., 2025; Shao et al., 2025). In this study, our aim is to integrate hotspot analysis with molecular dynamics analysis of calf chymosin (BtChy), with a specific emphasis on key motifs that impart excessive rigidity to the enzyme’s conformation. Our objectives encompass optimizing the enzymatic characteristics, augmenting its activity during milk curdling, minimizing activity loss during subsequent high-temperature treatment to terminate the reaction, and enhancing its suitability for the cheese-making process. To guarantee a sustainable and environmentally friendly enzyme production process, we screened for constitutive promoters and secretory signal peptides. Furthermore, we adopted the strategy of constructing concatemers of gene expression cassettes to boost enzyme production and obtain a high-yield chymosin strain that meets the requirements of the cheese industry.

## 2 Materials and methods

### 2.1 Molecular design and mutant construction of chymosin BtChy

The stability and activity of bovine chymosin B (PDB: 4AA8) were analyzed for hotspots using HotSpot Wizard 3.0 (Sumbalova et al., 2018). Five motifs, namely RIPLYKG, QAIGATQ, TQEPGDV, ACEGGCQ, and LGTPPQE, which potentially impact stability and introduce excessive rigidity to the enzyme structure, were identified for modification. The model of chymosin was extract from protein structure database PDA: 4AA8, and the 3-D structure of *κ*-casein was modeled by AlphaFold2 (Lyu et al., 2024). The molecular docking between chymosin and its substrate casein was performed using ZDOCK (Pierce et al., 2014). Molecular dynamics analysis of BtChy was conducted by using the Desmond V6.6 with optimized Lennard-Jones potential for solvation (OPLS4) force field (Schrödinger Inc., NY). Initially, a 1 nm cube reaction box was established containing 38,651 TIP3P water molecules, 11 sodium ions and pH8.0. This was followed by a 5 ns temperature equilibration (NVT) at 60°C and a 1 ns pressure equilibration (NPT). Subsequently, a 100 ns molecular dynamics simulation was performed using the NRI mathematical model to analyze residue dynamics in aqueous solvent system with OLPS force field. The simulation temperature condition for the empty protein is 338.15 K, and other conditions are the same as above description. During these simulations, the V-rescale method was used for temperature coupling, and the Berendsen method was employed for pressure coupling. The trajectory was analyzed using gmx trjconv, while RMSD and RMSF analyses were conducted using gmx rms and gmx rmsf software, respectively (Van Der Spoel et al., 2005; Vanommeslaeghe and MacKerell, 2012). The protein structure was displayed by PyMol software,^1^ and the hydrogen bond was plotted using ‌VinaLigGen method (Agrawal et al., 2024).

**Figure 1:**
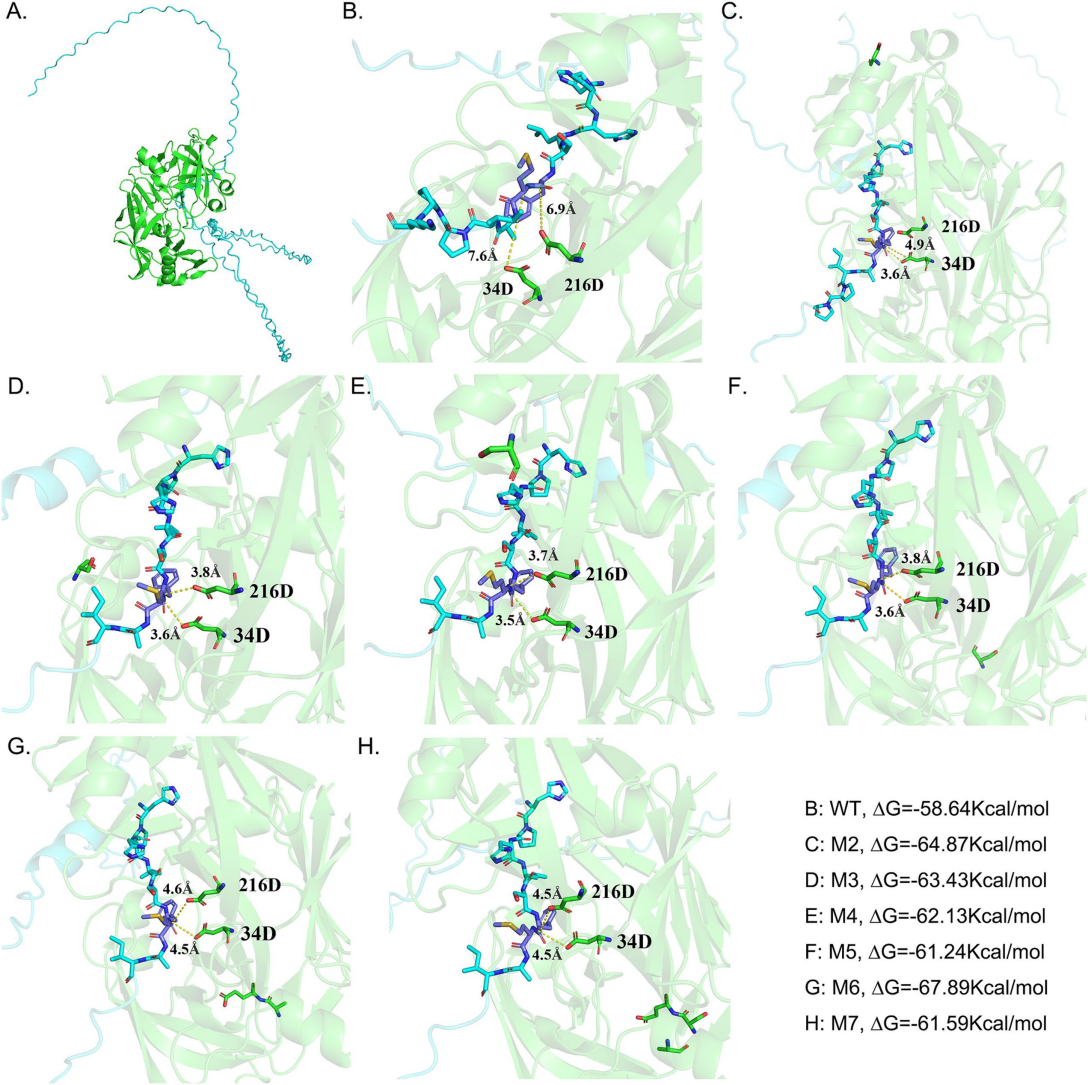
Displays the interactions between the amino acids in the active site of chymosin BtChy, its mutants, and the substrates. The residues located within a 4 Å radius adjacent to the cleavage site are highlighted. **(A)** The profile of the interaction between the chymosin and *κ*-casein. **(B–H)** The interaction details between the κ-casein and the active sites of wild type chymosin (WT) and the mutants M2, M3, M4, M5, M6, and M7, respectively. The mutant of M1 happened in pro-sequence of chymosin, and not be illustrated.

**Figure 2:**
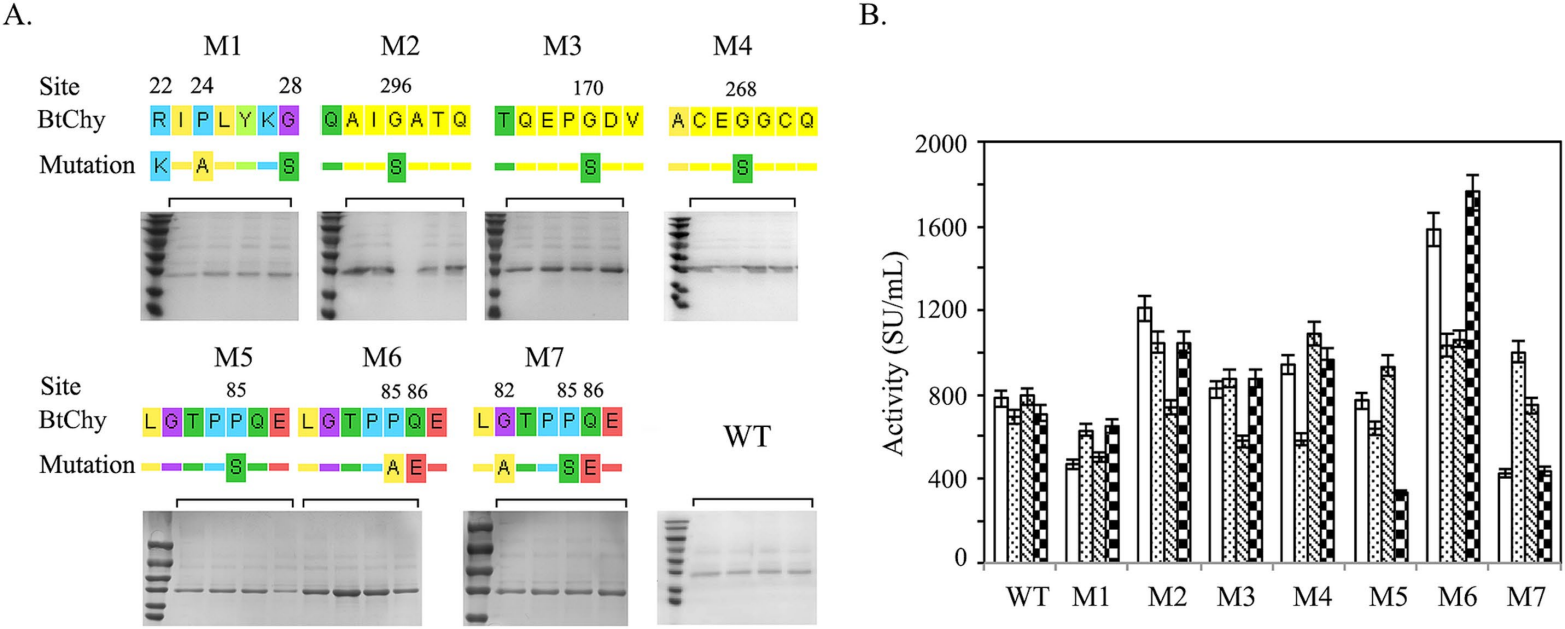
Point mutation of BtChy to improve its activity. **(A)** The diagram indicates the mutant sites and protein profiles of the recombinants checked by SDS-PAGE. **(B)** Chymosin activity of BtChy and its mutants.

Seven mutants designed as M1 (KIALYKS, R22K-P24A-G28S), M2 (QAISATQ, G296S), M3 (TQEPSDV, G170S), M4 (ACESGCQ, G268S), M5 (LGTPSQE, P85S), M6 (LGTPAEE, P85A-Q86E), M7 (LGTPPQE, G82A, P85S, Q86E) were constructed (Figures 1, 2A). The mutation sites were introduced into the gene through the inverse PCR method, with a summary of the mutation sites and PCR primers presented in Table 1. Using pKLAC_2_ as the vector (NEB #E1000S, NEB), recombinant plasmids carrying the mutated genes were constructed via one-step cloning technology.

**Table 1:** Primers for construction the BtChy mutants by reverse PCR.

Primers	Sequence 5′-3′
CHY^P85S^-F	GGGGACCCCGTCTCAGGAGTTCACCGTGC
CHY^P85S^-R	GAGACGGGGTCCCCAAGTAGATCTTCCCAAAG
CHY^P85A-Q86E^-F	GGGACCCCGGCTGAAGAGTTCACCGTGCTGTT
CHY^P85A-Q86E^-R	CTTCAGCCGGGGTCCCCAAGTAGATCTTCC
CHY^G82A-P85S-Q86E^-F	GATCTACTTGGCTACCCCGTCTGAAGAGTTCACC
CHY^G82A-P85S-Q86E^-R	CTTCAGACGGGGTAGCCAAGTAGATCTTCCC
CHY^R22K-P24A-G28S^-F	GCTGAGATCACCAGAATCGCTCTGTACAAATCTAAGTCTCTGC
CHY^R22K-P24A-G28S^-R	CTTAGATTTGTACAGAGCGATTCTGGTGATCTCAGC
CHY^G170S^-F	CCAGGAGCCATCTGACGTCTTCACCTATGCC
CHY^G170S^-R	GTCAGATGGCTCCTGGGTGCTCAGGCCTACTGT
CHY^G268S^-F	GTGGCCTGTGAGTCTGGCTGTCAGGCCATC
CHY^G268S^-R	CCAGACTCACAGGCCACAACCACACCGCTGATG
CHY^G296S^-F	CAGCAGGCCATTTCTGCCACACAGAACCAGTAC
CHY^G296S^-R	GCAGAAATGGCCTGCTGGATGTTCAAGATGTCGCTGC

### 2.2 Screening of constitutive promoters and construction of recombinant plasmids

Select five constitutive promoters, namely the glyceraldehyde-3-phosphate dehydrogenase gene (GAP), L-threonine 3-dehydrogenase gene 3 (TDH_3_), glycosyl phosphatidyl inositol (GPI)-anchored protein (GCW_14_) (Liang et al., 2013), phosphoglycerate kinase (PGK_1_), sorbitol dehydrogenase (SOR_1_) (Akentyev et al., 2023) to regulate the expression of the BtChy gene. The promoters were amplified from the yeast genome by PCR and then inserted into the pKLAC_2_ vector carrying the chymosin BtChy gene using one-step fusion cloning technology (Clontech, Mountain View, CA). The primers used for promoter amplification are listed in Table 2.

**Table 2:** Primers used to amplify the promoters used in this study.

Primers	Sequence (5′-3′)
pLAC-BtchyB-F	GATACCGTTTCGAATAATTAGTTGTTTTTTGATCTTCTCAAGTTGTC
pLAC-BtchyB-R	CAAAACACAATGAGATTTCCTTCAATTTTTACTGCTGTTTTATTCGCAG
Pgap-F	TAATTATTCGAAACGGGATCCTTTTTTGTAGAAATGTCTTGGTGTCC
Pgap-R	GAAGGAAATCTCATTGTGTTTTGATAGTTGTTCAATTGATTGAAATA
Ptdh3-F	ATTCGAAACGGGATCCTtctgccaagcacactcattcttctc
Ptdh3-R	GAAATCTCATTGTGTTTTGgtggaagcatagatgttcaaaagacgcaag
Pgcw14-F	GAAACGGTATCCTCCAGTGAGCTCGCTGGGTGAAAGCCAAC
Pgcw14-R	GAAATCTCATTGTGTTTTGTTTTGTTGTTGAGTGAAGCGAGTGACGG
Ppgk1-F	TTATTCGAAACGGGATCCTAGCGATATGGCACTAGTTGGGTATTCAAATAGTTG
Ppgk1-R	GAAGGAAATCTCATTGTGTTTTGGAGTGCTTCAACTGGAATTCCATC
Psor1-F	TAATTATTCGAAACGGGATCCTgtgttaaaaagttgtatattattaatg
Psor1-R	TGAAGGAAATCTCATTGTGTTTTGgttggataatagtgagtgtaatg

### 2.3 Screening of secretion signal peptides and their fusion expression with chymosin Btchy

The Btchy mutant with the highest enzymatic activity was used as the original gene for screening signal peptides. Nine signal peptides, full-length signal peptide of *α*-mating factor (α-MF), truncated α-mating factor (T-MF), α-amylase signal sequence (Amy), inulinase presequence (Inu), serum albumin signal (HSA), invertase signal sequence (Inv), lysozyme signal peptide (Lys), glucoamylase signal peptide (Glu), and killer protein signal peptide (Kil) were selected. Using primers containing signal peptide sequences, the fusion expression of signal peptides with BtchyB was constructed by the reverse primer method (Table 3). The PCR products were treated with Dpn I enzyme to eliminate the plasmid DNA template, and then ligated into the recombinant vector using the one-step cloning technique as described by the manufacturer (Clontech, Mountain View, CA). The positive plasmids were transferred into yeast competent cells by electroporation according to the protocol (NEB #E1000S).

**Table 3:** Primers for fusion expression the signal peptides with BtChy gene by reverse PCR.

Primers	Sequence 5′-3′
α-MF—F	cagcatcctccgcattagctctcgagaaaagagaggctgaag
α-MF—R	agctaatgcggaggatgctgcgaataaaacagcagtaaaaattgaag
Amy—F	ggtcttcaggtcgctgcacctgctttggctctcgagaaaagagagg
Amy—R	cagcgacctgaagaccgtacagaaacaaagaccaccaagcgaccatcgtttc
Glu —F	ctttgtctggtttggtttgtttggtttggctctcgagaaaagagaggctgaag
Glu —R	cagaacaaaccaaaccagacaaagccaacaaggatctaaaagacatcgtttcgaataat
Ser—F	tttgttgtttcttttctcttctgcttactctctcgagaaaagagagg
Ser—R	gaaaagaaacaacaaacagataaaggtaacccacttcatcgtttcgaataattagttg
Inu—F	cttgttgcttccattggcaggagtcagtgctctcgagaaaagagag
Inu—R	caatggaagcaacaaggagtatgctaacttcatcgtttcgaataattag
Inv—F	ctttccttttccttttggctggttttgcagccaaaatatctgcactcgagaaaagagag
Inv—R	caaaaggaaaaggaaagcttgcaaaagcatcgtttcgaataattagttgttttttg
Kil —F	gtattagttagatccgtcagtatattatttttcatcacattactacatctagtcgtagctctcgagaaaagagag
Kilr —R	ctgacggatctaactaatacttgggttggcttagtcatcgtttcgaataattagttgt
Lys—F	gttttggtcttgttgggattgactgctttgttgggtatctgtcaaggtctcgagaaaagagag
Lys —R	ccaacaagaccaaaacaagacacattgggtcgttcttacccagcatcgtttcgaataattagttg

### 2.4 Construction of a multi-copy recombinant expression plasmid for Btchy

**Figure 3:**
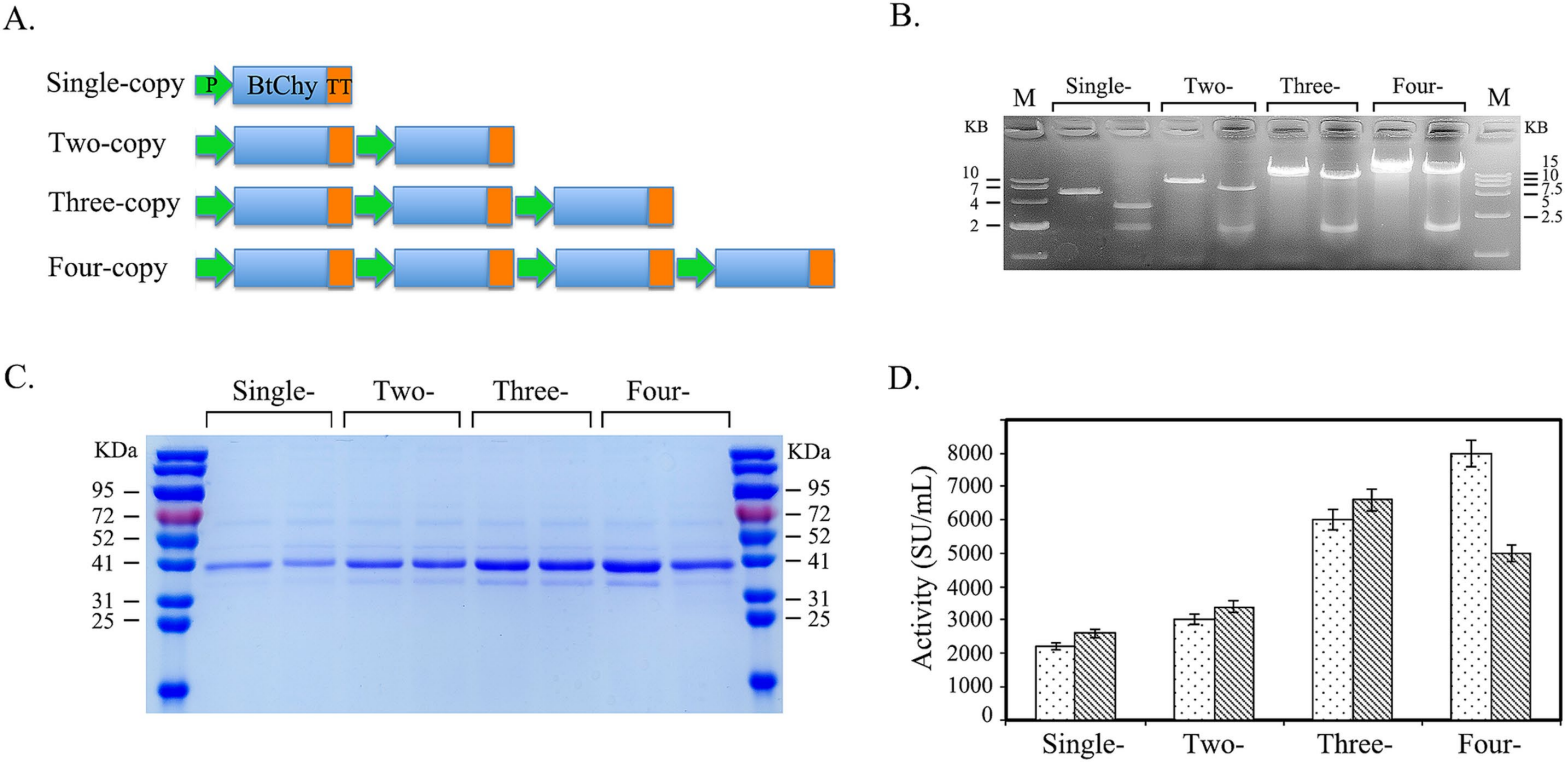
Improving the secretory expression of BtChy by increase the gene-dosage in the genome of Kluyveromyces lactis by *in vitro* constructing the concatemers of expression cassettes. **(A)** Schematic diagram of in vitro construction the concatemers of expression concatemers of BtChy. **(B)** Checking the size of the concatemers of expression cassettes. **(C)** SDS-PAGE checking the supernatant of the culture of BtChy recombinants. **(D)** The chymosin enzyme activity of the supernatant of BtChy recombinants carrying different copy number of expression cassettes.

The expression cassette comprising a strong constitutive promoter (P_TDH3_), the Inv signal peptide, and the mutated BtChy gene (M6) was constructed *in vitro* for tandem expression. The cassette ends were introduced with Xba I (C/TCGAG) and Sal I (G/TCGAC) isoschizomers. After double digestion, the expression cassette fragments were ligated together, forming hybrid sites in between, while the Xba I and Sal I sites were retained at the fragment ends, resulting in tandem constructs with two-, three-and four-copies of the expression cassette (Figure 3A).

### 2.5 Enzyme characteristics determination

The chymosin activity was assayed using the Arima K method. Briefly, milk powder was dissolved in HAc-NaAc buffer (pH 5.5) containing 50 mg/L CaCl_2_ to prepare a milk solution of approximately 10% (w/v). A 25 mL aliquot of the prepared milk solution was poured into a 50 mL test tube. The mixture was pre-incubated at 30°C for 10 min, and then 0.5 mL of diluted enzyme solution was added. The reaction mixture was immediately mixed and observed until the milk began to coagulate. The exact reaction time was recorded, and the enzyme activity was calculated based on this time. One Soxhlet Unit (SU) was defined as the amount of chymosin required to clot 1 mL of milk solution under above-described conditions.

Chymosin reactions were performed at temperatures ranging from 40, 45, 50, 55, 60, 65, 70, 75, to 80°C to identify the optimal temperature for the enzyme and to assess its chymosin activity. To determine temperature stability, the mutated chymosin was incubated at 65°C for 3 h, with residual enzyme activity measured at regular intervals thereafter.

## 3 Results

### 3.1 Hotspot scanning coupled with molecular dynamics analysis redesigned the key amino acids in chymosin

The process of curdling milk with chymosin to cause casein to coagulate and precipitate out of solution is a crucial step in cheese production. The *κ*-casein features a hydrophobic, negatively charged terminus. It forms a hairy surface on the micelles, causing them to repel each other and remain stable in a water-soluble state. Chymosin specifically cleaves the bond between Phe105 and Met106 of the hairy terminus of *κ*-casein by Asp 34 and Asp 216 in the activity site of enzyme through the dual acid theory, reducing the repulsive forces between the micelles (Figure 1A). This leads to the micelles sticking together, precipitating, and forming a curd. To enhance the activity of chymosin BtChy, a hotspot scan of the enzyme was conducted. Five motifs—RIPLYKG, QAIGATQ, TQEPGDV, ACEGGCQ, and LGTPPQE—were selected for modification. The Z-docking and LigPlots hydrogen band analysis among chymosin and κ-casein revealed that binding ∆G the of wild type was 58.64 kcal/mol, and the mutants except M1 were all higher than that, especial the M6 mutant has ∆G = 67.89 kcal/mol (Figures 1B–H). The distance between the active acids (34D, 216D) and cleave sites κ-casein revealed that in the wild type chymosin, the value were 7.6 Å and 6.9 Å. While in the mutants the distances were significantly short than that (Figures 1B–H). Furthermore, the number of hydrogen band between wild type enzyme and κ-casein were also less than the mutants (Supplementary Figures S1–S7).

**Figure 4:**
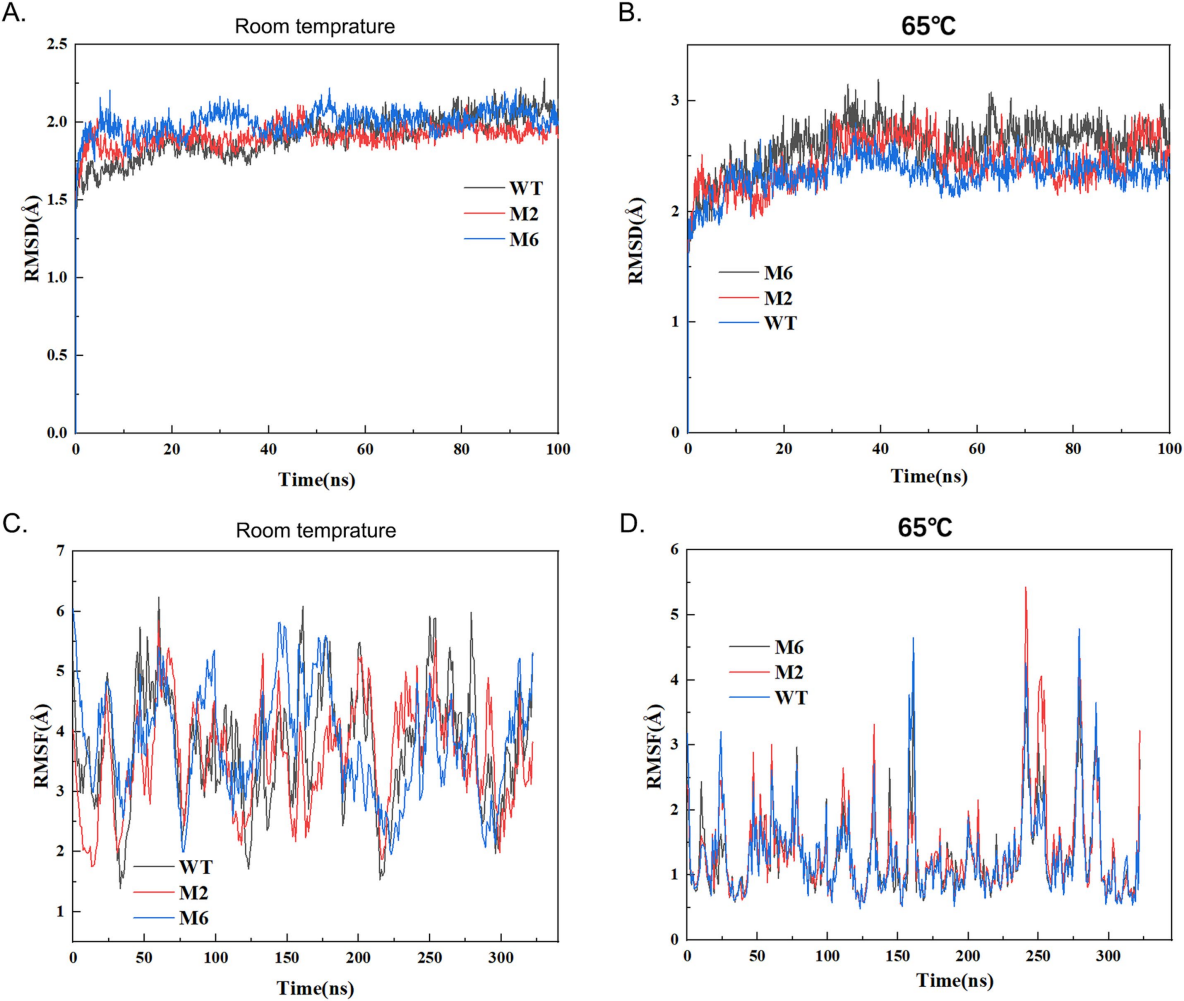
The energy variation RMSD and RMSF analysis of the wild type and mutant chymosin. The molecular dynamics simulation conditions are that the protein is placed in a 1 nm small cube box using an OPLS4 force field, and the water solvent model in the box uses TIP3P water molecule solvent; The system first uses the conjugate gradient method to minimize energy in 1000 steps; Under NVT conditions, the system is subjected to controlled heating from 0 to 303.15 K, with an integration step size of 1 fs and a duration of 100 ps; Then perform a 50 ps equilibrium simulation on the system for a duration of 100 ns. **(A,C)** RMSD and RMSF analysis of wild typy (WT) and mutant chymosin at room temperature. **(B,D)** RMSD and RMSF analysis of wild type (WT) and mutant chymosin at 65°C.

We further utilized Boltzmann machine learning direct coupling analysis (bmDCA) to scrutinize the correlations among these enzymes. The MD analysis was initially conducted under room temperature (Figures 4A,C). As revealed by figures, enzyme WT and mutants generally has flattened RMSD curves under room temperature, and the fluctuate RMSF curves, and revealed these enzymes were generally stable and active under room temperature. Notably, when temperature increased to 65°C, the mutant enzyme demonstrates a higher and more flattened RMSD line in comparison to the wild-type (WT) enzyme, suggesting potential decreased stability following the molecular design process (Figures 4B,D). Specifically, for the mutant M6 (P85A, Q86E), the RMSD and RMSF analysis highlights notable differences in the range of motion of atoms associated with enzyme activity, as compared to the wild-type (WT). These findings imply that the mutant’s increased molecular extensive motion of residues may contribute to its heightened activity toward substrates (Figure 4).

### 3.2 Molecular design improved the enzymatic properties of chymosin Btchy

According to the molecular dynamics analysis, seven mutants M1 (R22K, P24A, G28S), M2 (G296S), M3 (G170S), M4 (G268S), M5 (P85S), M6 (P85A, Q86E), and M7 (G82A, P85S, Q86E) were constructed (Figure 2). As depicted in the Figure 2, M1, featuring amino acid substitutions in the pro-sequence of chymosin, adversely affects the expression of BtChy. Conversely, M2, M3, and M4, which involve the substitution of glycine with serine, exhibit positive effects, with a notable increase in activity ranging from 20 to 50%. The presence of the double proline residues (P84 and P85) in the LGTPPQE motif may impart undue rigidity to the enzyme conformation, leading to a decrease in enzyme activity. To optimize the motif, mutants M5 (P85S), M6 (P85A, Q86E), and M7 (G82A, P85S, Q86E) were created. Among all mutants, M6 (P85A, Q86E) has boosted activity from 800 to 1800 SU/mL.

**Figure 5:**
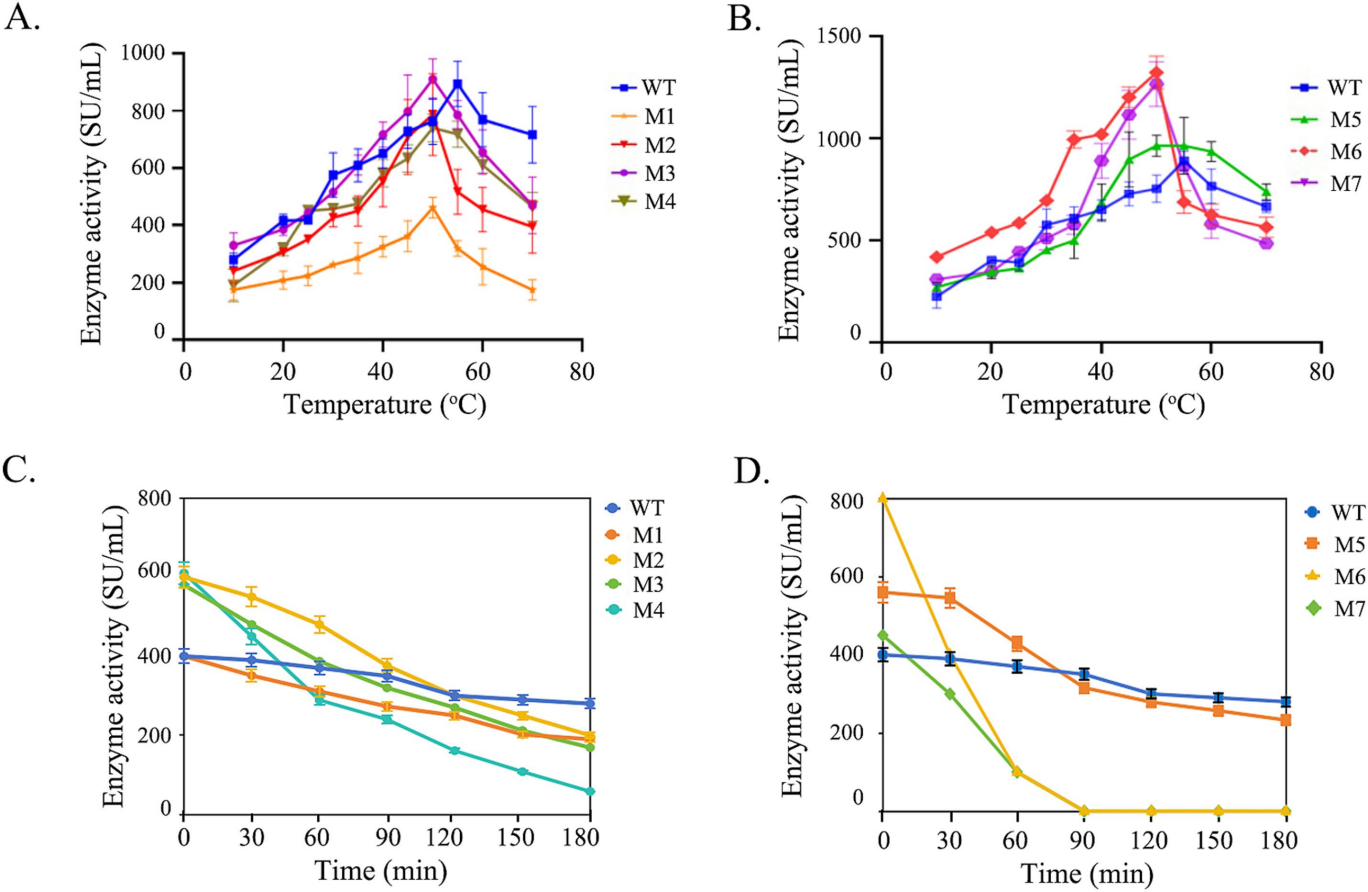
The optimal temperature of wild type and mutated BtChy. **(A,B)** The activity curves of enzymes incubated in different temperature. **(C,D)** The remaining activity when enzyme incubated under 65°C.

In the process of cheese making, curdling of milk occurs optimally within the temperature range of 30–33°C. Subsequently, a temperature of about 65°C is employed to inactivate chymosin, thereby terminating the coagulation reaction, eliminate the risk of the cheese becoming overly hard and bitter in subsequent manufacturing steps. The optimal temperature and thermostability profiles of both wild type chymosin and its mutants were analyzed (Figure 5). The wild-type chymosin (WT) exhibits an optimal temperature of 55°C, whereas all mutant variants display an optimal temperature of 50°C (Figures 5A,B).

After incubating the enzyme solution at 65°C, the residual activity was assessed at various time intervals (Figures 5C,D). The results indicate that the wild-type chymosin maintains stability with prolonged incubation time at 65°C. In contrast, the activity of all mutant variants decreased with extended heat treatment time. Notably, the activities of mutants M6 and M7 dropped sharply when the treatment time reached 30 min. After 1 h of heat treatment, almost no activity was detected in these mutants. Based on these experiments, mutant M6 exhibited superior enzyme activity and temperature sensitivity compared to the wild type, making it suitable for practical industrial applications.

### 3.3 Constitutive promoters enhanced the expression of chymosin BtChy

**Figure 6:**
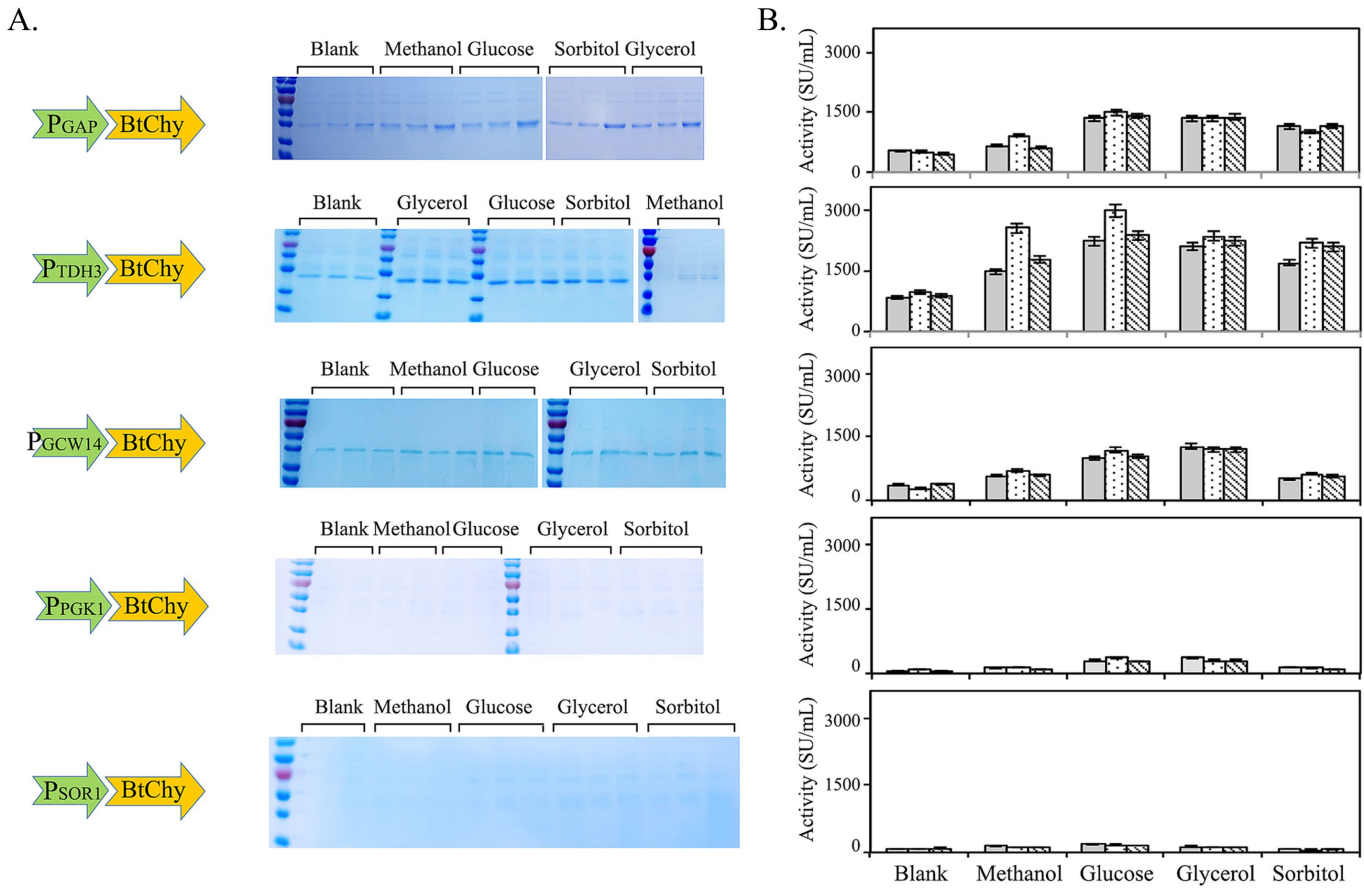
Promoter and culture carbon source screening for the expression of BtChy. Five constitutive promoters, P_GAP_, P_TDH3_, P_GCW14_, P_PGK1_, and P_SOR1_ were used to control the transcription of BtChy. The yeast recombinants were cultivated in YPD medium with glucose, glycerol, sorbitol and methanol as the carbon sources. **(A)** The profiles of BtChy expressed and controlled by constitutive promoters, and cultivated in different carbon sources. **(B)** The chymosin activity of the recombinants culture.

The utilization of constitutive promoters for regulating gene expression presents notable advantages, namely being eco-friendly, environmentally sustainable, and posing no safety risks during industrial production, particularly when the resultant enzyme products are destined for use as food additives, owing to the elimination of the need for inducers. In this investigation, five constitutive promoters, specifically P_GAP_, P_TDH3_, P_GCW14_, P_PGK1_, and P_SOR1_, were carefully selected to direct the expression of the chymosin gene (depicted in Figure 6). Additionally, the study employed diverse carbon sources, encompassing glucose, glycerol, sorbitol, and methanol, to assess their specific influences on the expression levels of the enzyme (illustrated in Figure 6). Evidently, the promoters P_GAP_ and P_TDH3_ demonstrated superior capacity compared to P_PGK1_, P_GCW14_, and P_SOR1_ in terms of driving the expression of the BtChy gene. Among the carbon sources tested, glucose emerged as nearly the most effective option, although glycerol also exhibited good performance. Overall, P_TDH3_ was identified as the optimal promoter, and glucose was found to have the most favorable impact on BtChy gene expression compared to the other carbon sources. Under these conditions, the enzyme activity attained a level of 2,700 SU/mL after 96 h of cultivation in a flask (as shown in Figure 6).

### 3.4 Identifying optimal signal peptides to enhance secretory expression levels of chymosin

**Figure 7:**
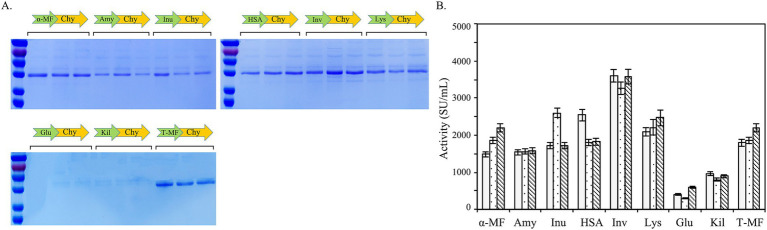
Signal peptide screening to improve the secretory expression of BtChy. The full-length signal peptide of *α*-mating factor and truncated (α-MF, T-MF), α-amylase signal sequence (Amy), Inulinase presequence (Inu), serum albumin signal (HSA), invertase signal sequence (Inv), lysozyme signal peptide (Lys), glucoamylase signal peptide (Glu), and killer protein signal peptide (Kil) were used. The protein profile in the culture were detected by SDS-PAGE **(A)**, and the activity of the recombinant protein were checked correspondently **(B)**.

To further streamline the enzyme purification process, this study undertook a rigorous screening to identify the optimal signal peptide for maximizing the secretory expression of BtChy in yeast, thereby enhancing its yield and facilitating downstream processing. Nine signal peptides, the full-length signal peptide of *α*-mating factor and truncated (α-MF, T-MF), α-amylase signal sequence (Amy), inulinase presequence (Inu), serum albumin signal (HSA), invertase signal sequence (Inv), lysozyme signal peptide (Lys), glucoamylase signal peptide (Glu), and killer protein signal peptide (Kil) were used (Figure 7A). As depicted in the figure, these signal peptides demonstrate differing abilities to facilitate enzyme secretion. Notably, the invertase signal sequence (Inv) proved to be the most effective signal peptide for enhancing the secretory expression of chymosin, resulting in yeast recombinants with an enzyme activity of 3,700 SU/mL (Figure 7B).

### 3.5 Optimizing expression by concatenating multiple BtChy expression cassettes

The expression cassettes, comprising the promoter (P_TDH3_), Inv signal peptide, and BtChy (M5), were concatenated *in vitro*. The resulting concatemers were verified through enzyme digestion and subsequently transformed into yeast to obtain recombinants with a higher copy number of the BtChy gene in their genome (Figures 3A,B). The expression profiles of these recombinants were analyzed (Figures 3C,D). As shown in the figure, the expression level increased correspondingly with the number of genes in the genome. Although variability in enzyme activity was observed among the recombinants carrying four-copy genes, the most productive strains belonged to this group. Notably, the enzyme activity of the four-copy recombinant reached 8,200 SU/mL, markedly higher than that of the single-copy strains (Figure 3).

### 3.6 Enhanced chymosin production through high-density cultivation

**Figure 8:**
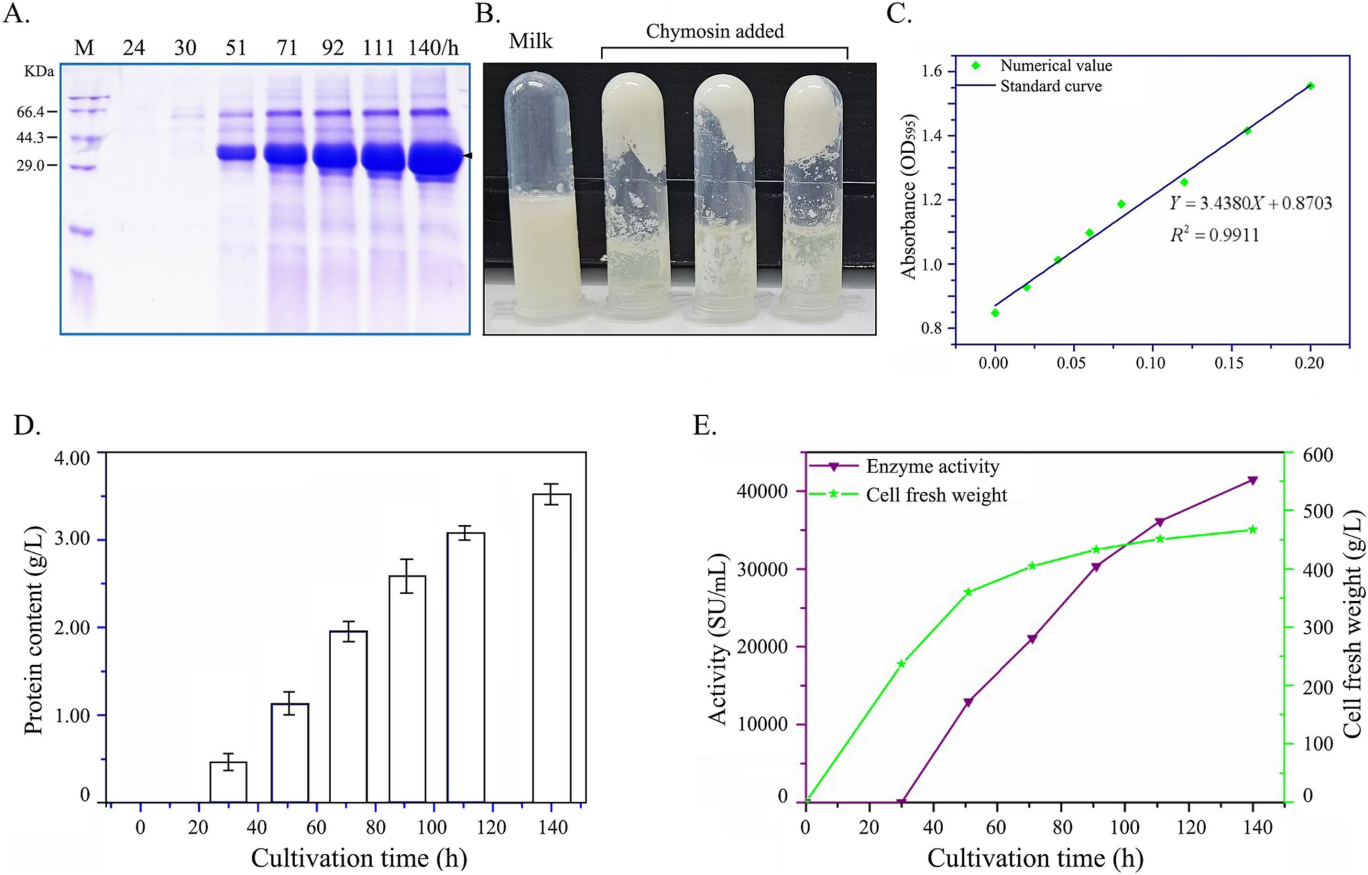
Production of chymosin BtChy in a 50-L bioreactor with high-density cultivation. **(A)** The protein profiles of supernatant checked by SDS-PAGE. **(B)** The curdling of milk after inoculates chymosin. **(C)** The protein standard curve for protein quantitation. **(D)** Protein content in the supernatant of culture. **(E)** The cell fresh weight of the culture and the enzyme activity measured.

High-density cultivation of chymosin recombinants was performed in a 5-L bioreactor, with measurements of protein content and enzyme activity in the culture supernatant taken at 24-h intervals (Figure 8). As illustrated in Figures 8A,B, the chymosin was effectively secreted by yeast and showed the milk curdling capacity. Meanwhile, the protein content in the culture gradually increased over time. At the 140-h time point, the protein content peaked at 3.6 g/L (Figures 8C,D). The fresh cell weight in the culture progressively climbed to 460 g/L, concurrent with the chymosin activity, as assayed by the Arima K method, steadily ascending to 42,000 SU/mL (Figure 8E). Notably, these metrics exhibited a significant enhancement of approximately 52.5-fold compared to the baseline levels observed with the original wild-type gene.

## 4 Discussion

### 4.1 Molecular design optimization enhanced the characteristics of chymosin

The traditional process of cheese preparation encompasses several steps: preparing milk, inoculating and curdling the milk with rennin (chymosin), cutting the curd, applying higher temperature treatment, separating cheese curds from whey, kneading the cheese mass, pressing, molding, and ripening (Brown and Pariser, 1975). The curdling step involves inoculating chymosin and allowing it to react at 30–32°C, causing casein to coagulate and precipitate out of the milk solution. Calf chymosin (BtChy), exhibits highly specific hydrolysis of the bond between Phe105 and Met106 of kappa-casein by a dual acid model (Langholm Jensen et al., 2013). This results in cheese with a more favored flavor compared to other sources of chymosin. Historically, calf-sourced chymosin has remained the most popular enzyme for this purpose.

**Figure 9:**
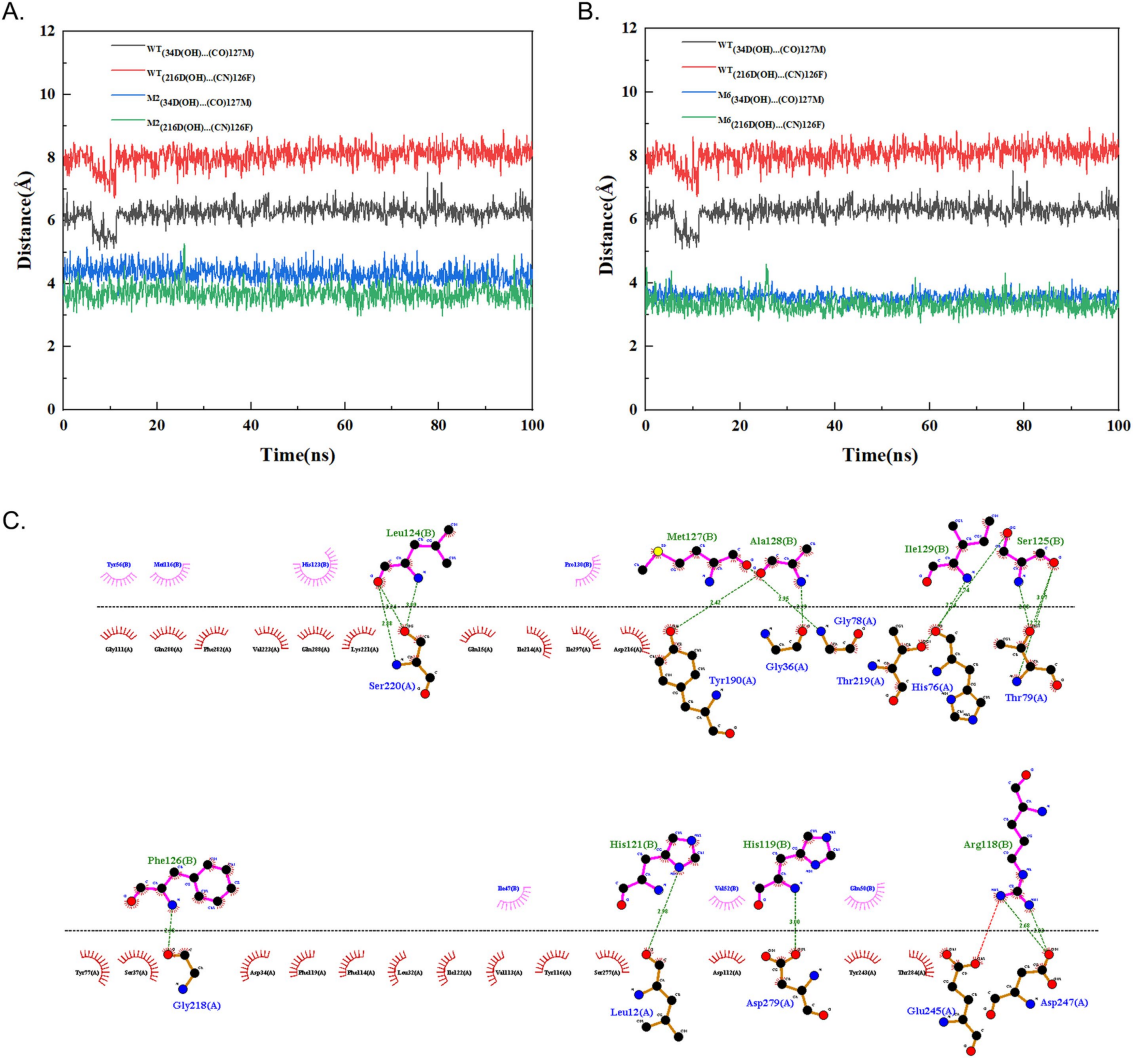
Molecular dynamics analysis of cleavage distance between chymosin and κ-casein according to the double acid reaction and the hydrogen band analysis. **(A)** Mutant M2 and wild type chymosin (WT). **(B)** Mutant M6 and wild type chymosin (WT). **(C)** The hydrogen band network between chymosin M6 and κ-casein.

To enhance the enzyme characteristics of BtChy, multiple strategies including hotspot scanning and molecular dynamics analysis were employed in its design. The HotSpot Wizard tool leveraged various protein engineering strategies to identify four categories of hotspots: functional, stability, and correlated hotspots (Sumbalova et al., 2018). While methods like FireProt (Bednar et al., 2015) offer similar functionality, they typically provide a list of promising sites without definitive guidance on specific amino acid substitutions. In contrast, our approach goes beyond merely listing potential sites by incorporating virtual screening and molecular dynamics analysis of the identified BtChy hotspots (Figures 1, 4). This facilitates the identification of key residues during conformational changes and offers profound insights into the mechanisms governing protein allostery (Zhu et al., 2022). The simulation reveals a steep decline in the RMSD curve comparing mutants to wild-type enzymes, with significant differences in vibrancy between them (Figures 1, 4). Notably, in the M6, which includes proline at positions 84 and 85, the double mutation M6 (P85A, Q86E) in motif LGTPPQE, results in a significant increase in the activity of the optimized enzyme to 1800 SU/mL, representing a 120% enhancement. Further analysis the distance and hydrogen band between enzyme and substrate *κ*-casein revealed than the mutants have short distance and more hydrogen bands than the wild type enzyme (Figures 4, 9; Supplementary Figures S1–S7). Thus, the reasons for the improve on the activity on the mutant enzyme might because enzyme bind more tightly to substrates.

The mutants, especially M6 was sensitivity to high temperatures, and will lose its activity after incubated at 65°C for half an hour. Therefore, the mutated enzyme, with its favorable characteristics, is more suitable for the cheese-making process compared to the wild-type enzyme. Certainly, the weaker thermostability of M6 might bring the trade-offs in enzyme shelf-life and robustness during storage and transport. We think the better conditions such as a cool environment in storage and transportation process will be facilitate to maintaining the stability of enzymes. In this study, the motifs abundant in glycine and proline, which may impart excessive rigidity to the enzyme conformation. As reported, the subamino ring in the proline side chain stabilizes the molecular conformation and also triggers ribosome stalling, reducing the speed of protein synthesis (Melnikov et al., 2016). By replacing the double proline, the activity and high-temperature sensitivity of enzymes have been improved.

### 4.2 Multiple strategies realize high-level and environmental-friend production of chymosin

The use of a constitutive promoter for gene expression offers advantages including being green, environmentally friendly, and safe for industrial production, particularly for enzyme products intended as food additives. In contrast, inductive promoters like AOX_1_, ADH_3_, and LAC_4_ require methanol, ethanol, or lactose for gene expression induction. Inducers such as methanol and ethanol may raise concerns regarding safety in production and food security (Mastropietro et al., 2021). In this study, we employed the constitutive promoters P_GAP_, P_TDH3_, P_GCW14_, P_PGK1_, and P_SOR1_ to facilitate the expression of the chymosin gene. As clearly depicted in Figure 6, the promoters P_GAP_ and P_TDH3_ exhibited superior capacity for expressing BtChy in comparison to P_PGK1_, P_GCW14_, and P_SOR1_, notwithstanding reports suggesting strong expression capabilities of the latter two for other genes (Liang et al., 2013; Akentyev et al., 2023). Several carbon sources were evaluated for their impact on chymosin expression, and glucose emerged as the optimal choice. This indicates that glucose not only supports the growth of yeast but also potentially lacks a feedback inhibition effect on chymosin expression (Takpho et al., 2018). Consequently, the P_TDH3_ promoter paired with glucose as the carbon source constitutes the best combination for enhancing BtChy expression. Subsequent experiments involving the screening of eight signaling peptides revealed that the invertase signal sequence (Inv) exhibited the strongest secretory expression of chymosin (Figure 7).

Increasing the gene dosage in host cells is an effective strategy to elevate the expression level of the target gene (Lloyd et al., 2018; Vegesna et al., 2019). In the *K. lactis* expression system, it is possible to obtain recombinant strains carrying multicopy genes through multiple insertions into the genome via homologous recombination events (*K. lactis* Protein Expression Kit, NEB, Massachusetts). However, screening for multicopy-gene recombinants from a yeast recombinant library that contains numerous false-positive transformants is a time-consuming process. *In vitro* construction of tandem expression cassettes for the gene, followed by their direct integration into the yeast genome, constitutes a more efficient approach to obtaining multicopy-insertion recombinants (Dagar and Khasa, 2018; Yang et al., 2019). To generate a multi-copy gene insertion strain, we created a series of concatemers *in vitro* (Figure 3). As the number of genes in the genome increased, the expression level correspondingly enhanced, with the enzyme activity of the four-copy recombinant reaching 8,200 SU/mL—substantially higher than that of single-copy strains.

High-density cultivation of the recombinants carrying the four-copy optimized chymosin gene was then conducted in a 5-L bioreactor. As depicted in Figure 8, after approximately 140 h of cultivation, the chymosin activity reached 42,000 SU/mL. This level was notably higher than the chymosin activity achieved through fusion-expression with glucoamylase in *Aspergillus awamori* (Ward et al., 1990). Additionally, it surpassed the activities reported using the alcohol-yeast *Komagataella phaffii* (*Pichia pastoris*) and expression driven by the strong promoter AOX_1_ (Noseda et al., 2016; Ali Hanoğlu et al., 2021).

## 5 Conclusion

In conclusion, the strategies integrating hotspot scanning and molecular dynamics analysis have effectively optimized the enzymatic characteristics of chymosin BtChy, which significantly enhanced its suitability for cheese production. The chymosin-producing strains, which possess optimized constitutive promoters, signal peptides, and multiple copies of the chymosin genes integrated into the yeast genome, present a green and environmentally friendly approach for chymosin production. Following high-density cultivation, the chymosin activity reached a level of 42,000 SU/mL, representing a significant 52.5-fold increase compared to that of the original wild-type chymosin gene.

## Data Availability

The datasets presented in this study can be found in online repositories. The names of the repository/repositories and accession number(s) can be found in the article/Supplementary material.

## Footnotes

^1^ https://www.pymol.org/
