# Supplementary material for: Multiple strategies were adopted to optimize the enzymatic characteristics and improve the expression of bovine chymosin BtChy in Kluyveromyces lactis for cheese production
Source: Front Microbiol. 2025 May 29;16:1605229. doi: 10.3389/fmicb.2025.1605229 (PMC12158995; doi:10.3389/fmicb.2025.1605229)
Supplement: Supplementary file 1 [file Presentation_1.pptx]

## Slide 1
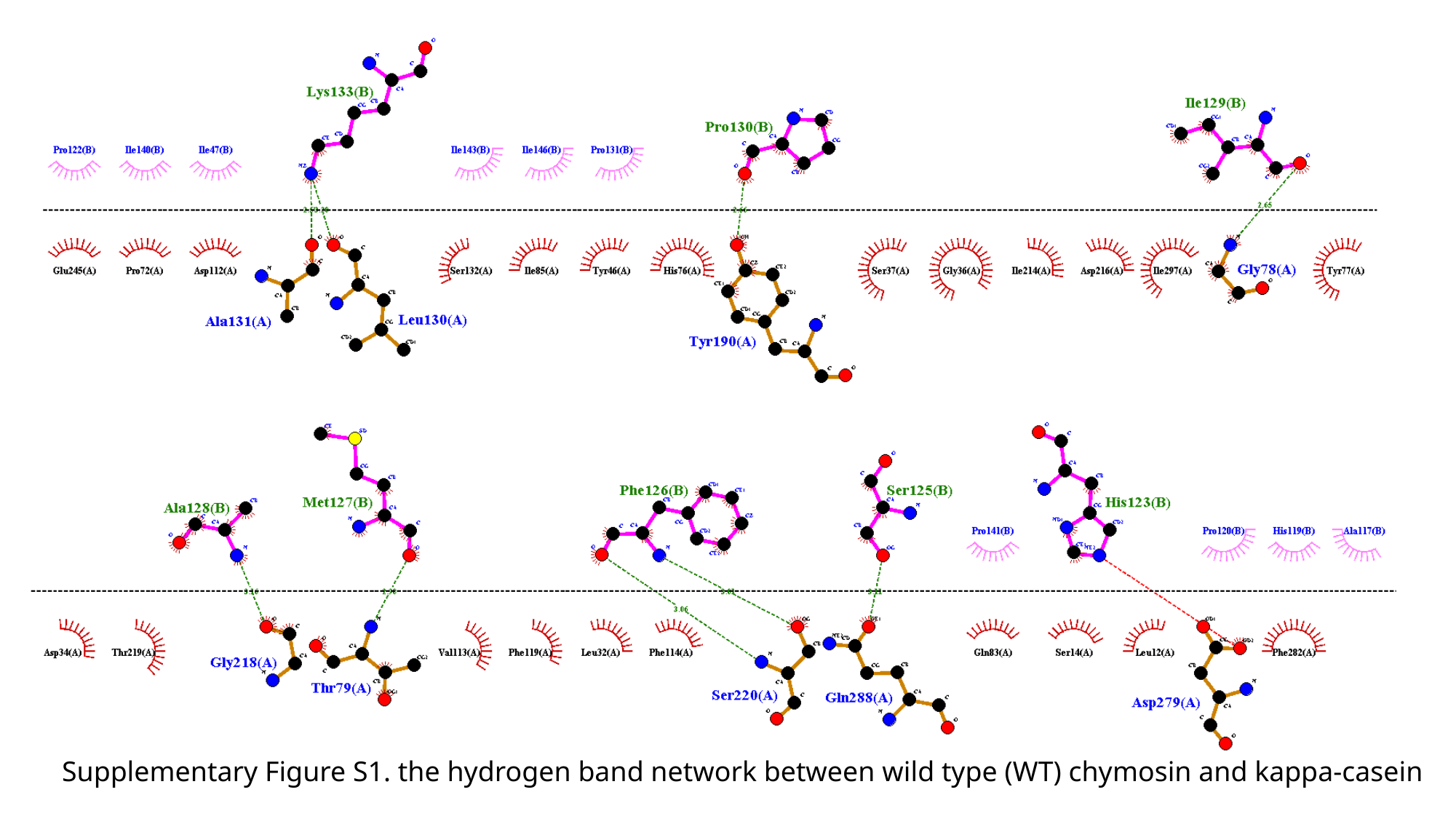

Supplementary Figure S1. the hydrogen band network between wild type (WT) chymosin and kappa-casein

## Slide 2
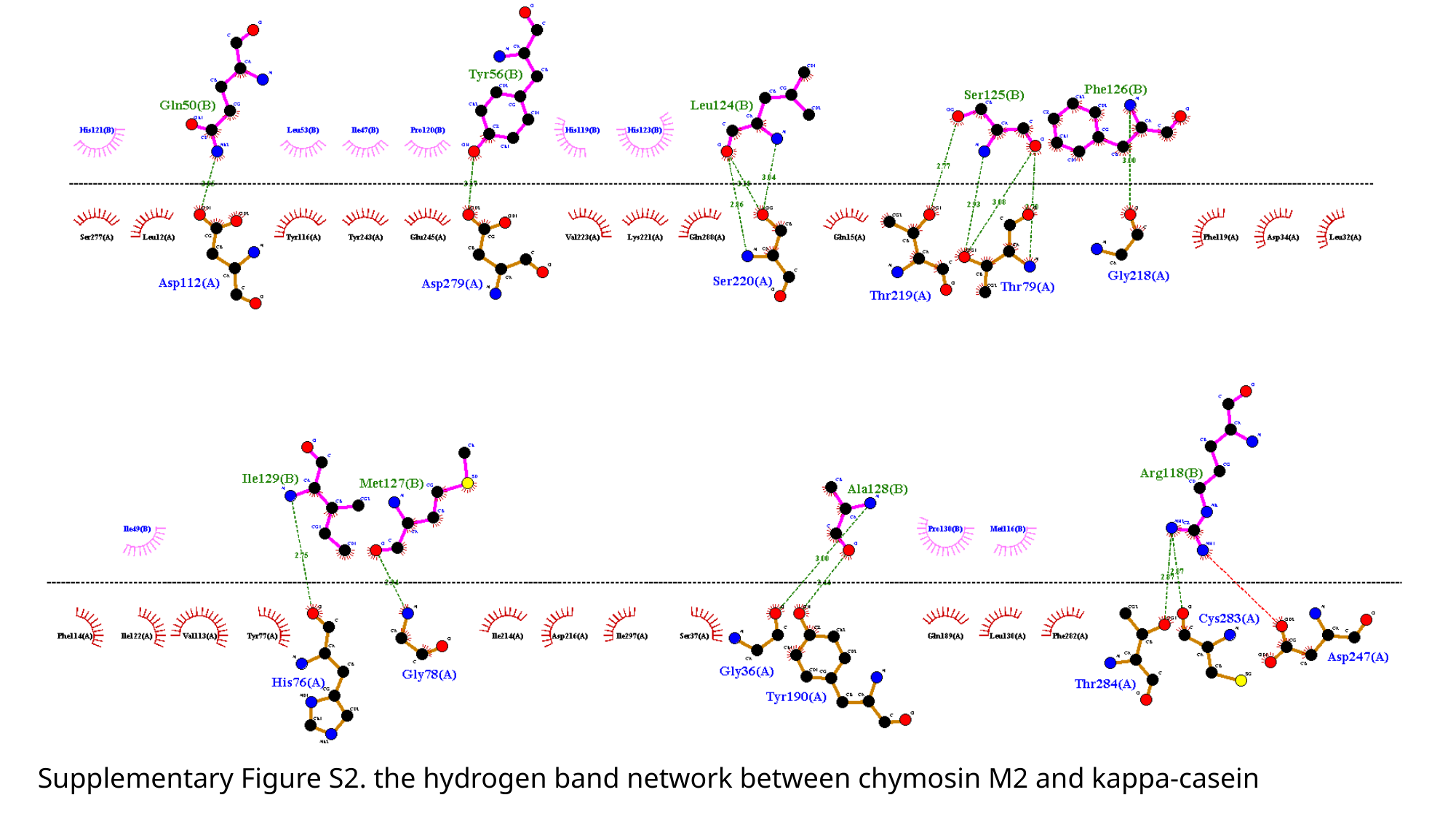

Supplementary Figure S2. the hydrogen band network between chymosin M2 and kappa-casein

## Slide 3
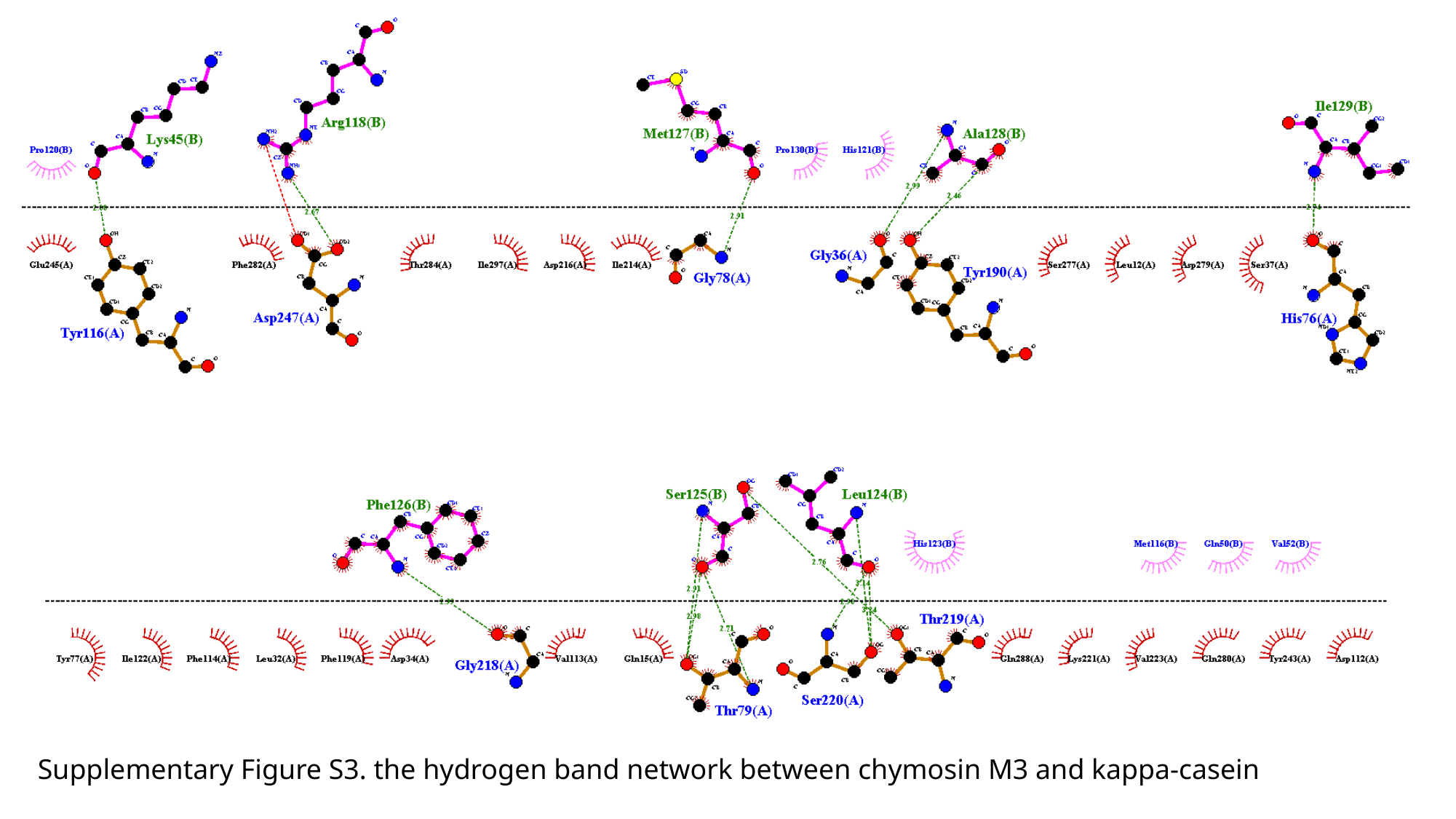

Supplementary Figure S3. the hydrogen band network between chymosin M3 and kappa-casein

## Slide 4
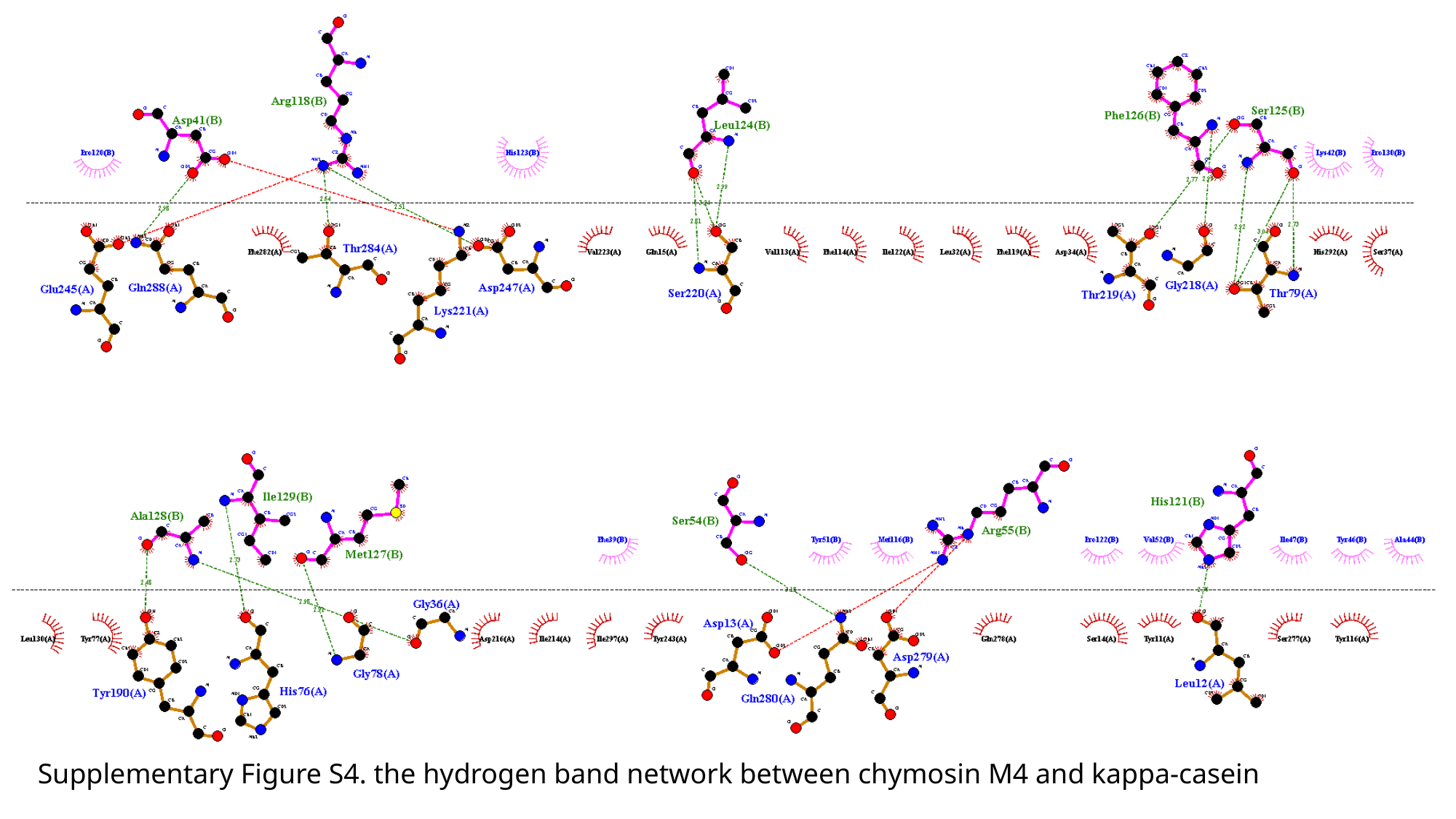

Supplementary Figure S4. the hydrogen band network between chymosin M4 and kappa-casein

## Slide 5
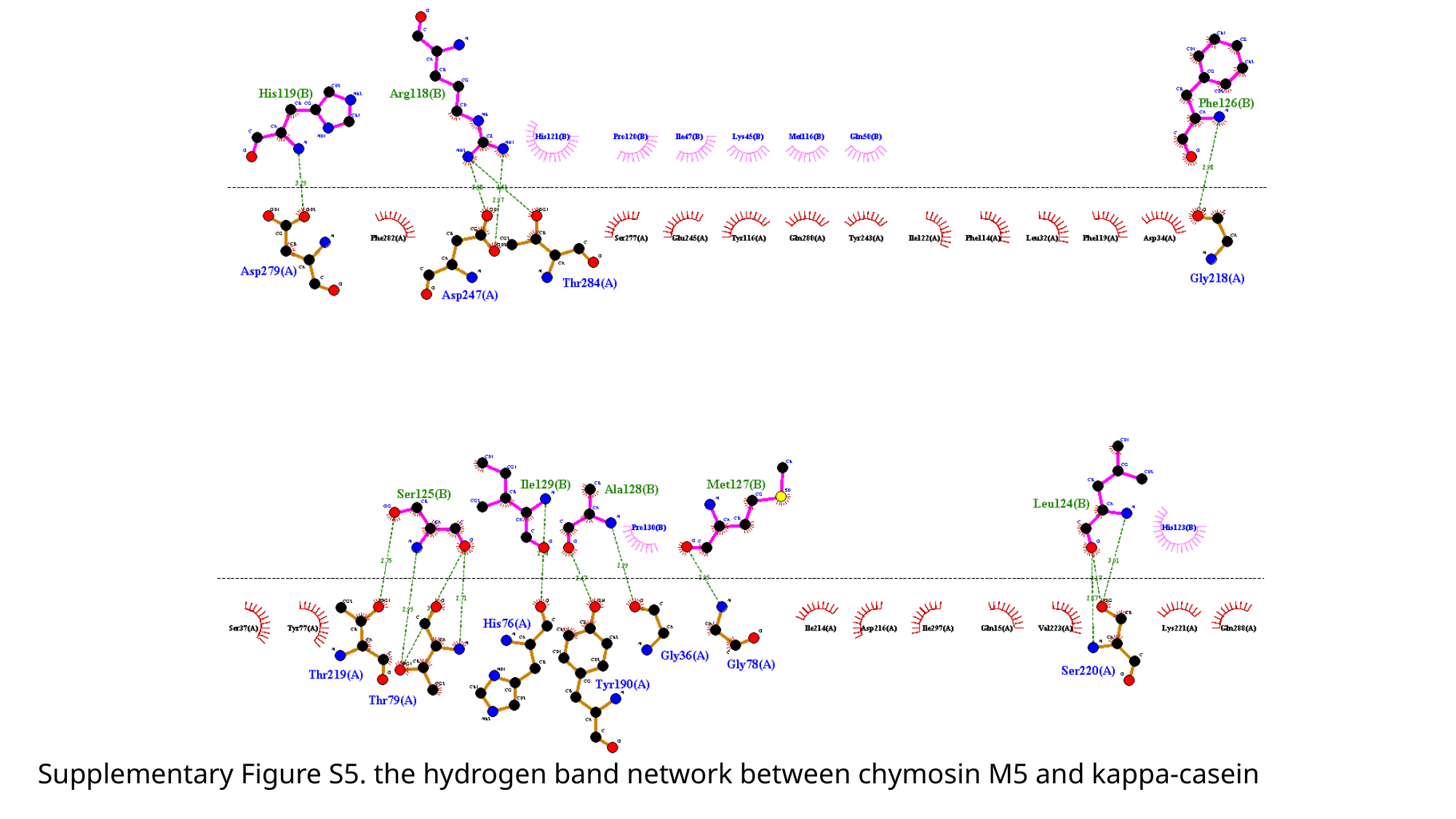

Supplementary Figure S5. the hydrogen band network between chymosin M5 and kappa-casein

## Slide 6
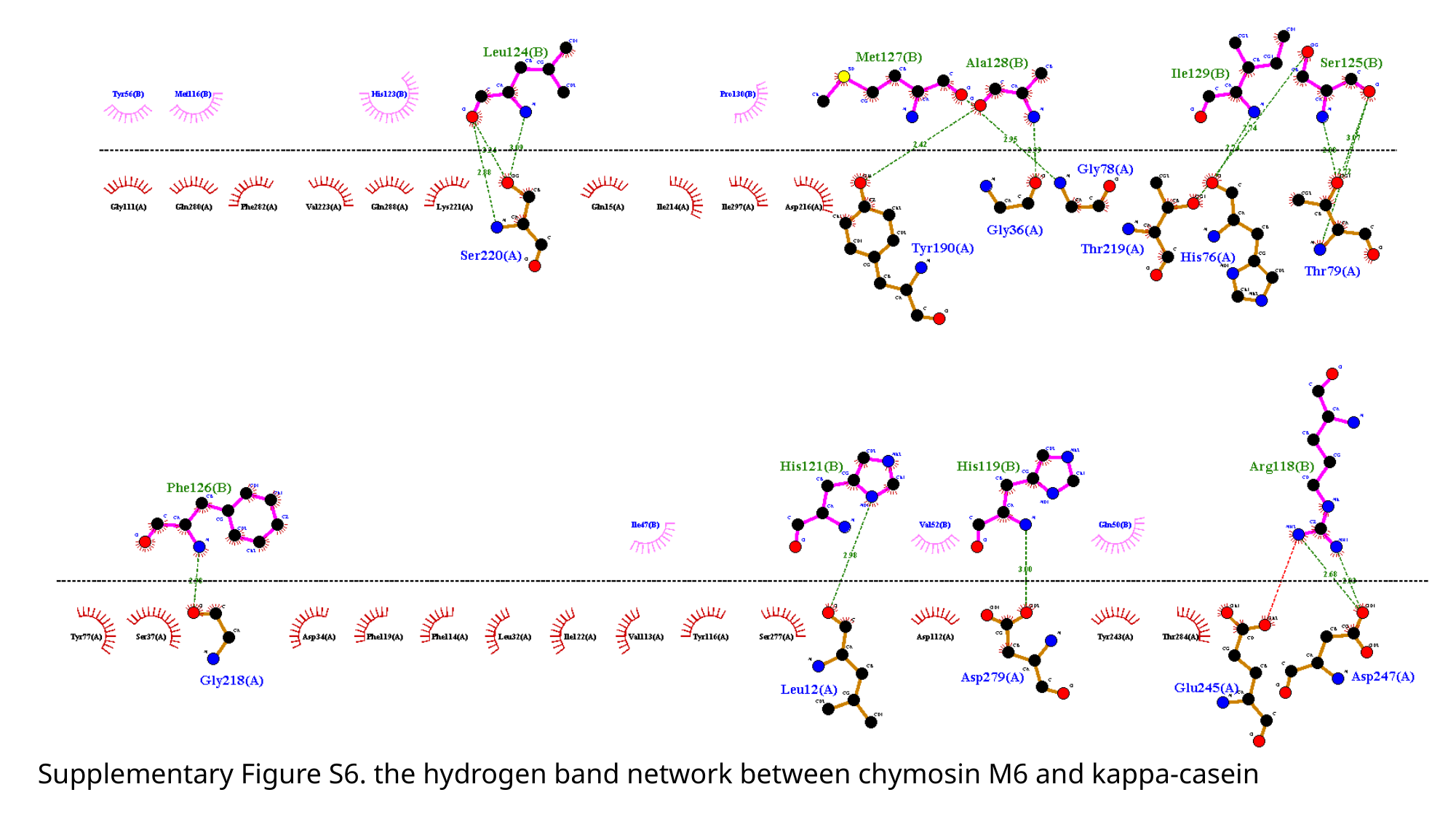

Supplementary Figure S6. the hydrogen band network between chymosin M6 and kappa-casein

## Slide 7
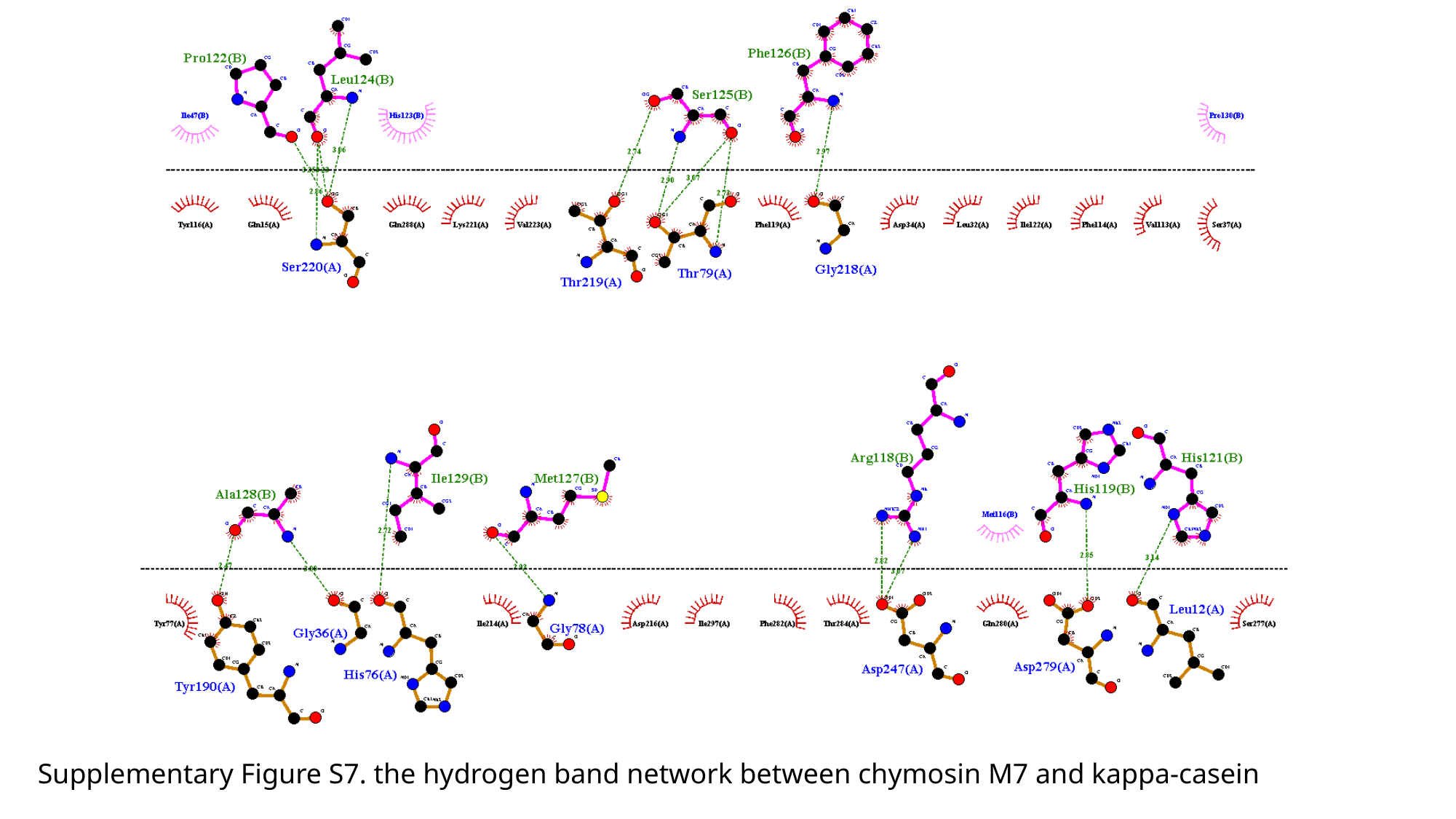

Supplementary Figure S7. the hydrogen band network between chymosin M7 and kappa-casein
